# Patient Mealtime Experience: Capturing Patient Perceptions Using a Novel Patient Mealtime Experience Tool

**DOI:** 10.3390/nu15122747

**Published:** 2023-06-14

**Authors:** Kate Furness, Melina Harris, Annie Lassemillante, Stephen Keenan, Natasha Smith, Katherine J. Desneves, Sam King

**Affiliations:** 1 Department Sport, Exercise and Nutrition Sciences, School of Allied Health, Human Services and Sport, La Trobe University, Bundoora, VIC 3086, Australia; 2 School of Health Sciences, Swinburne University of Technology, Hawthorn, VIC 3122, Australia; 3 Austin Health, Nutrition and Dietetics Department, Division of Allied Health, Heidelberg, VIC 3084, Australia

**Keywords:** patient experience, food services, dietetics, malnutrition, hospitals, rehabilitation, quality

## Abstract

Introduction: The aim of this study is to describe the mealtime experience using the qualitative components of the Austin Health Patient Mealtime Experience Tool (AHPMET) to complement the quantitative findings of this tool. Methods: A multiphase, cross-sectional study was undertaken across all sites of Austin Health (Victoria, Australia) between March 2020 and November 2021. Patient mealtime experience was measured using the AHPMET. Descriptive statistics and a deductive thematic analysis approach described the patients’ mealtime experiences. Results: Questionnaire data were collected from 149 participants. Patients were most satisfied with staff interactions, and least satisfied with dimensions of food quality, specifically, flavour, presentation, and menu variety. Clinical symptoms, nutrition impact symptoms and the patient’s position were barriers to consumption. Discussion: Food quality was perceived as the poorest aspect of patient satisfaction with the hospital foodservice, particularly flavour, presentation, and menu variety. Future foodservice quality improvements must prioritise improving food quality to have the greatest impact on patient satisfaction. While clinical and organisational systems have a role in improving mealtime experience and oral intake, communicating patient perceptions of the mealtime experience is critical for responding to current perceptions of hospital food quality. Conclusion: Mealtime experience in the hospital has a significant impact on oral intake and patients’ wider perception of hospital services. Questionnaires have been used to capture patient satisfaction with foodservice in the hospital; however, no comprehensive questionnaires including qualitative questions that capture the broader mealtime experience have been validated across different hospital settings. The tool developed through this study can be implemented in any acute and subacute health service to provide feedback and improve the mealtime experience of patients. This has the capacity to improve mealtime intake, mitigate malnutrition, and improve quality of life and patient outcomes.

## 1. Introduction

### 1.1. Background 

Malnutrition, described as both a cause and result of disease [1], is associated with adverse patient outcomes such as increased length of stay, risk of falls and infections, higher in-hospital mortality, readmission rates and healthcare costs, and poorer health outcomes [2]. Research in Australian hospital settings suggests approximately 31–35% of inpatients are malnourished [2,3] with higher prevalence in certain patient populations (e.g., oncology, rural patients) [4]. Hospital foodservices are essential in optimising patients’ nutritional intake and status through the provision of food, beverages, and nutrition support [5,6]. Poor food intake is associated with increased risks of malnutrition and mortality, and is common in hospital patients due to personal, clinical, and/or foodservice factors [1]. Foodservice factors such as meal quality, appearance and the mealtime environment are recognised as causes of inadequate nutrition in patients [7]. 

A consumeristic relationship between patients and healthcare services has grown over recent decades, with greater patient expectations regarding quality of services [7,8]. Patient experience is more broadly described as a reflection of the quality of healthcare [9], whereby positive experiences are associated with improved clinical outcomes and reduced healthcare costs [10]. In relation to patients’ interactions with food/nutrition, the concept of patient mealtime experience (MTE) is complex and associated with multidimensional factors including food quality and sensory aspects [7,11]; subjective aspects of satisfaction [12,13] and acceptability [5,14,15,16]; mealtime environment [16,17]; interactions with staff [15,17,18,19], volunteers and visitors [14,20]; meal timeliness and accuracy [7,15]; and choice and variety [13,21]. Any one of these factors could conceivably impact patient food intake, but these factors are often studied in isolation [20,22]. Nonetheless, there will inevitably be interactions between each of these factors, meaning that access to a tool which measures the MTE construct as a whole is critical [1].

Questionnaires are commonly used to measure patient experiences with healthcare service provision [5,7], including several validated tools for measuring patient experience of hospital foodservices [23,24]. Most commonly in Australia, the Acute Care Hospital Foodservice Patient Satisfaction Questionnaire (ACHFPSQ) [24] developed from the Wesley Hospital Foodservice Patient Satisfaction questionnaire (WHFPS), is utilised. While this tool has proven to be reliable, accurate and valid in hospital patients, both in Australia and internationally [25], this tool does not include qualitative patient MTE data, or explicitly assess patient satisfaction with the menu ordering system, and factors affecting nutritional intake [24]. Expanding patient MTE assessment to include these aspects may help provide a more in-depth characterisation of patient MTE and/or identify further areas for exploration.

A validated tool, expanded to include more domains of patient MTE and allowing for qualitative feedback, would allow opportunities for the ongoing monitoring and evaluation of how foodservice operations are perceived by consumers in hospital foodservice. Results from this tool could then rapidly identify areas for quality improvement and be used to guide strategic planning of these improvements which aim to improve both patient MTE and clinical outcomes, including malnutrition. 

### 1.2. The Study

Aim: The aim of this study is to describe the mealtime experience of patients in one metropolitan Australian health service using the qualitative components of AHPMET to complement the quantitative findings of this novel tool. 

## 2. Methods

### 2.1. Study Design 

This multiphase, cross-sectional study was undertaken across all sites of Austin Health (Heidelberg, Victoria, Australia) between March 2020 and November 2021. The theoretical framework underlying this study was person-centred care (PCC), described as a key attribute of high-quality health services [26]. The National Quality and Safety Health Service Standards explicitly mentions PCC as a core aspect of best practice healthcare provision, recognising that patients should be “partners in their own care” [27]. PCC is an organisation-wide priority in Austin Health’s 2018–2022 Strategic Plan [28], and is particularly relevant in ensuring patient preferences are integrated into hospital foodservice planning and delivery.

### 2.2. Study Setting and Sampling 

Austin Health incorporates a large tertiary acute hospital (Austin Hospital, AH), a subacute, aged-care and rehabilitation setting (Heidelberg Repatriation Hospital), and a dedicated rehabilitation setting (Royal Talbot Rehabilitation Centre). The tool was pilot-tested on the acute population, but due to COVID-19, restrictions data could only be collected in the subacute/rehabilitation population. Convenience sampling was used to recruit participants in all phases. 

### 2.3. Inclusion/Exclusion Criteria

Adult inpatients who had received meals for at least one full day (i.e., breakfast, lunch, dinner), or carers or family members of patients who met these criteria, were considered eligible. Exclusion criteria were patients less than 18 years old, non-English speakers, those not receiving meals (i.e., nil by mouth or fasting), those admitted under the intensive care unit, brain disorders, acquired brain injury or palliative care units, diagnosed or suspected COVID-19, admission diagnosis of mental illness, or patients identified by nursing staff as inappropriate for participation based on medical symptoms or condition during data collection. 

### 2.4. Data Collection 

There were six phases of the overarching project. Literature reviews, including grey literature and peer-reviewed journals, were completed in each phase to inform the development of the tool. Phases 1 and 2 involved interviews with Austin Health staff (foodservices, clinical, consumer engagement) and patients to develop a novel tool for capturing patient MTE at Austin Health. In Phase 3, the tool was pilot-tested with consumers for feedback, tool refinement was undertaken based on this feedback and baseline data were collected. Phases 4–6 involved further data collection of patient MTE. These data will be used to establish the validity and reliability of the tool for the Austin Health patient population (to be reported on separately). 

The final Austin Health Patient Mealtime Experience Tool (AHPMET, Supplementary File S1) is a 32-item questionnaire categorised into four domains: Food Quality, Environment, Staff Interactions and Food Ordering System. Eighteen questions require responses according to a six-point Likert scale (1 = never to 5 = always, with N/A coded as 0) and one question (regarding overall satisfaction with meals) has a three-point Likert scale (yes/sometimes/no). Short answer questions were included to offer participants opportunities to provide qualitative feedback, as recommended by previous research [28]. The AHPMET also contains five questions to capture the demographic data of participants, including type of hospital diet and admission length. 

All patients who met inclusion criteria were considered potential participants. Researchers liaised with nursing staff and/or ward dietitians to identify suitable patients. Eligible patients were approached in-person or via telephone call (due to COVID-19 hospital restrictions) and asked to be involved in the study. To minimise inter-person variability, a standardised verbal script was used to explain key information about the study and deliberately included an explanation of the term ‘mealtime experience’ to ensure participant understanding, based on recommendations from the literature [19]. Participants were provided with information about the purpose of the study and their rights in participating, with verbal and written consent obtained. Participants were offered a choice of completing questionnaires verbally with researchers (in-person or via telephone), independently via provided printed copies or electronically using a QR code link. No identifying data were collected.

All researchers contributed to data handling and cleaning. Data collected were digitally recorded in cloud-based spreadsheet files, accessible only by the research team. Numerical coding for quantitative, categorical data were completed at the time of digital data entry. To ensure rigour, cross-checking of raw data entries was performed and any discrepancies between hard-copy and digital raw data were corrected.

## 3. Ethical Considerations

The overall project was a collaboration between Austin Health Nutrition and Dietetics Department and Swinburne University of Technology’s Master of Dietetics course. Ethics approval for all phases of the project was granted by Austin Health HREC (Reference Number: HREC/61358/Austin-2020).

## 4. Data Analysis

Analysis of patient MTE data included descriptive analysis of quantitative data, and thematic analysis of qualitative data. SPSS software (version 28) was used for quantitative statistical analyses, whilst excel spreadsheets were used for thematic analysis of qualitative data.

Quantitative data were analysed descriptively. For continuous variables, normality testing was undertaken using the Shapiro–Wilk test for normality, with age and length of stay being found to be non-normal. All questionnaire data from Likert scales are presented using frequencies, as per previous recommendations [29], while continuous variables are presented using median and range, and mean and standard deviation. Categorical data (health service site, gender, medical unit, diet code and dining environment) are presented as frequencies. 

Thematic analysis of short answer questionnaire responses was conducted by two researchers according to the Framework Method [30]. A deductive thematic analysis approach was applied, whereby each researcher independently generated codes, themes and sub-themes for comments pertaining to each domain of the AHPMET. Consensus of final themes, sub-themes and codes was achieved by discussion between the researchers.

## 5. Results 

A total of 149 participants completed the AHPMET (48% male, 52% female) (Table 1). The median age was 77 (range 19–101) years, and median length of stay was 19 (range 1–270) days. The majority (75%) of participants consumed their meals in an individual or shared room, while the remainder (25%) consumed their meals in a shared dining room. Only two participants were included from Site 3 due to limited access to the acute site due to COVID-19 restrictions.

## 6. Qualitative Findings

### 6.1. Food Quality: Variety and Perception

The themes of food perception and variety dominated responses in the Food Quality domain (Table 2). Comments regarding food perception predominantly related to the taste/flavour and appearance of food, while the theme of variety largely related to the variety of meals throughout a day and across the menu cycle, and identified improvements to focus on including more fresh foods, culturally diverse staple foods and variation in cooking methods. 

### 6.2. Environment: Atmosphere, Functionality, and Clinical Symptoms 

Three themes emerged from the Environment domain: atmosphere, functionality, and clinical symptoms. This domain identified factors that inhibit mealtime intake and are a deterrent to the mealtime environment, including sensory aspects of the environment, other patients, accessibility and functionality of the mealtime setup and dining area, and clinical impact symptoms. Facilitators to mealtime/nutritional/food intake and a pleasant MTE were also identified, such as socialisation during meals and a mealtime environment that facilitates such interaction. Additionally, the available furniture used during mealtimes affected intake and/or comfort. 

### 6.3. Staff Interactions and Assistance: Manner

The theme of staff manner largely consists of patient perception that the staff are ‘friendly’, ‘polite’ and ‘go out of their way’ to assist during mealtimes. Interestingly, the need for staff delineation was highlighted, whereby knowledge of staff roles may assist in clarifying what patients should expect from each role. This was identified through patients’ responses, such as, ‘Patient Services Assistants (PSAs) won’t help me with the packaging’ (female, 85 years), and the suggestion that it ‘could be better if staff had different uniforms’ (female, 70 years). 

### 6.4. Food System Ordering: Service Accuracy, Menu Navigation and Meal Timing 

Service accuracy, menu navigation and meal timing arose under the Food Ordering System domain. Prominent issues included receiving incorrect meals or incorrect menu items, the illogical order of the paper menu, and reliance on staff to assist with completing the paper menu. Meal service times were commonly perceived as too early, particularly for lunch and dinner; however, meal service was considered punctual. 

### 6.5. Overall Satisfaction: Foodservice Quality 

The overarching theme identified in response to further comments about the mealtime experience was foodservice quality. Most responses related to food quality specifically noted the enjoyment of meals, as well as the need for greater variety, freshness, and flavour. Further comments comprised patients’ perception of the overall foodservice quality and experience, and whether this met their expectations and standards, as well as comments giving an indication to alter the timing of meal provision.

### 6.6. Overarching Themes: Differences between Sites and Patient Expectations 

Throughout all mealtime experience domains, two prominent themes emerged: the differences between sites, and patient expectations. Patients identified differences, primarily in food quality, between the three Austin hospital sites, referring to inconsistent food quality and differing availability or variety of food between sites, such as, ‘Variety is better at the Talbot compared to Austin’ (male, 65 years). Patient expectations of hospital food appeared to influence or determine patient perception of hospital food, particularly following the sentiment that patients expect hospital food to be of poor quality prior to their admission. This poor expectation was met or remained unchanged, ‘hospital food not good…don’t like hospital food’ (male, 70 years), or was exceeded to the surprise of patients, ‘it’s pretty good for hospital food (female, 46 years)’, and ‘found the service of meals up to my standard and quality of food, good quality’ (male, 83 years).

## 7. Quantitative Findings

Responses from the completed AHPMET (*n* = 149) are presented in Table 3. The Food Quality responses most frequently rated as positive were 6d (meal serve size being adequate) and 6e (meal served at a suitable temperature), with 92% and 83% of patients respectively rating these as often or always occurring. Conversely, the Food Quality responses most frequently rated as sometimes, rarely, or never related to patient satisfaction with food quality (6a) were the presentation of the meals (6f) and taste and flavour of the food (6g). With regards to the eating Environment, all questions were rated by most participants as never, rarely, or sometimes affecting their mealtime experience. Staff Interactions and Assistance were frequently rated positively, with each question being rated as often or always by over 87% of participants, and this was similar for the Food Ordering System questions, with each question rated as often or always by at least 70% of participants. Just over half of the participants indicated that their meals had been enjoyable overall, while only a small number indicated they did not find their meals enjoyable at all. 

## 8. Discussion 

The AHPMET captured and described the full breadth of the complex and multifaceted factors influencing patient mealtime experience at Austin Health. This is the first tool to broadly incorporate more qualitative aspects of MTE. Staff interactions, the physical environment, meal size and meal temperature were rated highly by patients, while satisfaction with the quality of food, including presentation, taste and flavour appeared to be the least favourably rated aspect of the MTE. The qualitative findings provide rich details on the aspects of MTE that contribute to patients’ satisfaction or dissatisfaction, therefore allowing for targeted quality improvement initiatives to be implemented and then further evaluated over time. 

Themes that emerged through thematic analysis overall reflected patient satisfaction in the four dimensions of mealtime experience. Food quality was the poorest performing domain of food services, observed in quantitative data and thematic analysis at both sites. This could be due in part to subjective individual patient preference of the diverse hospital population. Patient perception of hospital food quality is similarly reflected in other studies, where both the expectation gap of food quality and food quality issues contribute to poor satisfaction [5,14,26]. Patient comments regarding lack of variety were commonly related to menu repetition for long-stay patients, which is consistent with the known length of stay seen in the present rehabilitation setting. Stronger dissatisfaction with food quality at RTRC potentially highlights the effect of the foodservice model on food quality.

Features of the physical environment, including noise, visitors and other patients, room surroundings and ambience, interruptions by hospital staff and smells or odours were not commonly reported to affect food intake in the study. While mealtime interruptions have been described previously as prominent barriers to intake in the acute setting [15,18], this appears to be less of a factor in rehabilitation settings where the majority of this population was sourced [31]. However, we must acknowledge that interruptions, particularly by visitors, would have been significantly lower due to COVID-19 restrictions. These factors may help explain the present results given the small proportion of acute patients.

In contrast to the physical environment, clinical factors appeared to have a greater impact on patient’s nutritional intake. Nutrition impact symptoms, other clinical symptoms and physical limitations were factors shared in quantitative and qualitative questionnaire responses. Busy rehabilitation schedules causing fatigue, medical conditions or surgery impacting intake, and uncomfortable sitting position were additionally highlighted in patient comments. Similar factors were identified by Naithani et al. [32] where physical limitations during mealtimes, uncomfortable positions and rushed mealtimes were common. Physical inaccessibility to food has been identified elsewhere as a prominent barrier to intake [32]; however, clinical and nutrition impact factors affecting intake have not been previously described in other mealtime experience studies related to intake barriers. Patient experience tools measuring mealtime barriers further identify age as a predictor of physical access to food being a barrier [33], which may be more relevant to the geriatric population at HRH in this study. 

Patient experiences with staff were overwhelmingly positive in this study. Staff were found to be the most positively perceived aspect of hospital foodservices, congruent with other studies using the ACHFPSQ and interviews [7,19]. This was consistent in both interactions and assistance aspects of the domain, and positive patient comments regarding staff related to ‘friendliness’, being ‘nice’, going out of their way to assist and expressing concern. 

The overall patient experience with the Food Ordering System was positive for meal timing, accuracy of meals received, and courtesy of staff. However, qualitative responses to the Food Ordering System predominantly related to meal timing being too early, particularly lunch and dinner, and insufficient time between meals. Many patients acknowledged their understanding of the timing-related constraints of hospital food services. A smaller number of patients described receiving the wrong order or missing items from their meal. 

## 9. Implications for Practice

Patient feedback obtained in this study provides insights into patient perceptions of the food quality improvements needed. Previously studied interventions for improving patient satisfaction and food intake have focused on patient-centred foodservice models; however, specific innovations for improving taste, flavour, meal presentation and variety are lacking [34]. Increasing the use of herbs and spices is a cost-effective strategy for improving the flavour of meals as well as creating distinguishable taste between meals, addressing the needs of the diverse population and [35] alleviating the potential for taste fatigue in the hospital. Improving menu variety within existing foodservice models may be more challenging for healthcare services; however, prioritisation needs to be placed on developing strategies for increasing menu variety and repetition in response to the needs of long-stay patients in rehabilitation settings. Patients’ dissatisfaction with meal presentation, particularly the colour of meals identified in this study, may be further directions for research. Improving presentation may be achieved through colour, such as use of coloured vegetables, cooking methods that maintain produce colour, and the use of sauces in mixed dishes [10]. Enhancing the accessibility of food by patients with regard to their physical limitations is also a key concern that could be addressed through training and the enhanced role of responsibility of foodservice staff and nursing assistants.

## 10. Strengths and Limitations 

While this study provides important information on the MTE of hospitalised patients not captured previously, there were a number of limitations associated with this study. Firstly, the validity and reliability of the tool utilised has not yet been established. Validation against the ACHFPSQ, as well as internal validity and reliability testing, are currently being conducted. Still, the ability of the AHPMET to capture characteristics of the MTE validly and reliably is currently unclear. Furthermore, the AHPMET was developed somewhat based on the ACHFPSQ, which primarily targeted the acute setting, a population under-represented in the current study. It is therefore possible that some of the questions included may have been less relevant or appropriate for the population studied. Nonetheless, the development of the tool incorporated feedback from dietitians and patients across both acute and subacute sites, which may help mitigate this. Finally, while the addition of qualitative data helped triangulate and add weight to the quantitative results, timely analysis of these data may be difficult for health services in practice. 

## 11. Conclusions

This study expands the scope of tools that are available to effectively measure mealtime experience. The AHPMET captures a broad range of mealtime experience factors, including food quality, the environment, clinical barriers to nutritional intake, staff interactions and assistance, and the food ordering system. Food quality is fundamentally a priority to health service organisations, clinicians, food services and, most importantly, patients in improving patient satisfaction, quality of life and health-related outcomes. Innovative food quality improvement initiatives are required with a focus on improving presentation, taste and flavour of meals, and increased menu variety, particularly in long stay hospital settings. The tool adds distinct value to current measures of mealtime experience, with the inclusion of some unexplored factors of MTE.

## Figures and Tables

**Table 1:** Characteristics of the sample.

Characteristics			
		Frequency (*n* = 149) (%)	Median (IQR)
Age (years)		-	77 (19)
Length of stay (days)		-	19 (44)
	Site 1 HRH	-	11 (23)
	Site 2 RTRC	-	28 (70)
	Site 3 AH	-	47.5 (-)
Participants per site	Site 1 HRH	97 (65)	
	Site 2 RTRC	50 (34)	
	Site 3 AH	2 (1)	
Sex	Female	78 (52)	
	Male	71 (48)	
Medical Unit	Site 1: Geriatric	57 (38)	
	Site 1: Rehabilitation	33 (22)	
	Site 1: Surgical	4 (3)	
	Site 1: Orthopaedics	1 (0.7)	
	Site 2: Mixed rehabilitation	27 (18)	
	Site 2: Spinal rehabilitation	20 (13)	
	Not specified	7 (5)	
Diet code	Regular	94 (63)	
	Diabetic	24 (16)	
	Regular easy to chew *	16 (11)	
	High energy high protein	5 (3)	
	Other	10 (7)	
Dining environment	Shared dining room	37 (25)	
	Individual room/bed area	39 (26)	
	Shared room/bed area	73 (49)	

* Includes diabetic, short-term diet change, and texture-modified “regular easy to chew” codes. AH = Austin Hospital, HRH = Heidelberg Repatriation Hospital, RTRC = Royal Talbot Rehabilitation Centre.

**Table 2:** Thematic analysis.

Domain	Responses (*n*)	Theme	Definition	^ Code Frequency: Theme Perceived Positively	Exemplar Quote	^ Code Frequency: Theme Perceived Negatively	Exemplar Quote
FOOD QUALITY	22	Food perception	Patient perception of the sensory and technical aspects of meals. Sensory aspects encompass the flavour, smell, and appearance; and technical aspects, include temperature, serving size, quality, freshness and nutritional value of meals.	87	‘Excellent: vegetables cooked to perfection. Size of meals is perfect, and desserts are perfect.’ (male, 77 years, LOS 35 days)	114	‘Meals have no flavour, not to my liking; sometimes it doesn’t look very nice or appetising at all, but it tastes nice it’s just not appealing when you look at it.’ (female, 77 years, LOS 52 days)
		Variety	Availability of menu options suitable to the patients’ dietary and cultural needs, meal choice and repetition of the menu cycle.	21	‘Food is really good, big variety, they provide minced meat which is easier to chew and it’s very efficient.’ (male, 93 years, LOS 28 days)	62	‘Lack variety, reheated meals not appetising, same menu, too often. Nothing new.’ (male, 44 years, LOS 84 days)
ENVIRONMENT	11	Atmosphere	The sensory and social aspects of the mealtime environment, including noise, lighting, odours, temperature, mealtime interruptions and socialisation.	9	‘Was able to choose who I got to eat with and talk which was good.’ (male, 77 years, LOS 13 days)	22	‘The structure of the room makes it really noisy and loud—the high ceilings and big openness echoes noises—loud chattering and loud banging and crashing of the dishes in the kitchen and plates being cleared—makes it hard to hear, not peaceful.’ (male, 40 years, LOS 165 days)
		Functionality	The utility, practicality, and accessibility of the mealtime environment, including furniture, the mealtime setup and dining spaces.	8	‘Eat meals sitting in chair for comfort.’ (male, 78 years, LOS 3 days)	13	‘Sometimes there isn’t enough aisle space for wheelchairs or walkers once people are seated and eating.’ (female, 45 years, LOS 14 days)
		Intrapersonal factors	Clinical, medical, psychological, or personal factors that affect a patient’s ability or desire to eat.	1	‘In general, I am eating more in hospital than I would if I was at home—because at home if I’m busy e.g., in the garden sometimes I would skip a meal. Whereas in hospital I am eating all my meals.’ (female, 77 years, LOS 52 days)	23	‘My migraines sometimes affect how much I eat—pain. tiredness, smells, odours, nausea/vomiting, loss of appetite sometimes impact on my eating because of my migraines. If I’ve done a lot that day—e.g., physio, pool, I can be very tired and that affects how much I eat.’ (male, 66 years, LOS 90 days)
STAFF INTERACTIONS AND ASSISTANCE	87	Manner	The staff nature including verbal and non-verbal interactions, and attitude in providing mealtime assistance.	76	‘The staff are really helpful and take care of me. They set me up for mealtimes well and provide assistance when needed.’ (female, 49 years, LOS 3 days)	11	‘The staff are not personable and unsocial/anti-social.’ (male, 94 years, LOS 12 days)
FOOD ORDERING SYSTEM	76	Accuracy	The accuracy of meals delivered, or menu items received compared to what has been ordered.	2	[I want to] ‘Eat later, I understand people have their meals early. All meals received as ordered.’ (male, 83 years, LOS 4 days)	10	‘Not getting food ordered. Not getting the right food. Getting random food.’ (male, 66 years, LOS 182 days)
		Ordering navigation	The ability to read and order from the menu, including functionality of the menu, accuracy of meal descriptions and assistance received in ordering meals.	3	‘The staff help fill out paper menu (because can’t use hand)’. (female, 92 years, LOS 3 days)	7	‘The people collect my menu always not coming back and they don’t collect my menu.’ (female, 35 years, LOS 109 days)
		Timing	Appropriateness of mealtimes, spacing between meals, timing of ordering, and punctuality of meal service.	4	‘Breakfast 8am, lunch 12pm, dinner 5pm—usually spot on time.’ (female, 91 years, LOS 41 days)	35	‘I sometimes order 3 days in advance and don’t remember what I ordered. I’m not even sure if I want it that far in advance.’ (male, 94 years, LOS 24 days)
OVERALL SATISFACTION	108	Foodservice quality	The patient perception of the quality of meal provision and the overall foodservice experience.	46	‘Place to be if you’re going to be anywhere. Meals are fantastic and always on time.’ (male, 91 years, LOS 56 days)	41	‘Change/update menu more often, more variety, improve quality; add more fresh foods.’ (male, 19 years, LOS 30 days)

Footnotes: ^ code frequencies are a count of codes that appear within in each theme. LOS: length of stay.

**Table 3:** Descriptive statistics of Test 1 AHMPET item responses (*N* = 149).

Item		Not Applicable *n* (%)	Never *n* (%)	Rarely *n* (%)	Sometimes *n* (%)	Often *n* (%)	Always *n* (%)	*n*
Food Quality								
6a	How frequently have you been satisfied with the quality of the food you have received at Austin Health?	0	5 (3.4)	18 (12.1)	45 (30.2)	45 (30.2)	36 (24.2)	149
6b	Have the meals offered been appropriate for your beliefs or needs? (e.g., religious, cultural, vegan)?	24 (16.1)	8 (5.4)	6 (4.0)	17 (11.4)	18 (12.1)	76 (51.0)	149
6c	Has there been variety in your meal choices?	2 (1.3)	11 (7.4)	13 (8.8)	26 (17.6)	36 (24.3)	60 (40.5)	148
6d	Has the serving size of your meals been adequate?	1 (0.7)	3 (2.0)	0 (0.0)	8 (5.4)	22 (14.9)	114 (77.0)	148
6e	Have the meals been served at a suitable temperature?	0	6 (4.0)	2 (1.3)	18 (12.1)	29 (19.5)	94 (63.1)	149
6f	Have the meals looked appetising when they were presented?	0	16 (10.7)	8 (5.4)	40 (26.8)	40 (26.8)	45 (30.2)	149
6g	Has the taste and flavour of the meals been to your liking?	0	21 (14.1)	12 (8.1)	41 (27.5)	33 (22.1)	42 (28.2)	149
Environment Do the following factors affect the amount of food you eat during mealtimes?								
10a	Noise ^	2 (3.4)	108 (72.5)	19 (12.8)	12 (8.1)	3 (2.0)	5 (3.4)	149
10b	Visitors and/or other patients ^	11 (7.4)	107 (71.8)	12 (8.1)	11 (7.4)	4 (2.7)	4 (2.7)	149
10c	Room surroundings (e.g., layout of the room, furniture, lighting, ambience) ^	5 (3.4)	108 (72.5)	15 (10.1)	10 (6.7)	6 (4.0)	5 (3.4)	149
10d	Interruptions by hospital staff (e.g., wanting to speak to you or give you treatment) ^	3 (2.0)	84 (56.4)	29 (19.5)	23 (15.4)	7 (4.7)	3 (2.0)	149
10e	Smells and odours ^	2 (1.3)	106 (71.1)	19 (12.8)	15 (10.1)	4 (2.7)	3 (2.0)	149
Do the following aspects affect the amount of food you eat during mealtimes?								
12a	Loss of appetite ^	1 (0.7)	49 (32.9)	22 (14.8)	43 (28.9)	14 (9.4)	20 (13.4)	149
12b	Nausea and/or vomiting ^	4 (2.7)	101 (67.8)	17 (11.4)	20 (13.4)	3 (2.0)	4 (2.7)	149
12c	Pain ^	4 (2.7)	71 (47.7)	26 (17.4)	24 (16.1)	14 (9.4)	10 (6.7)	149
12d	Tiredness ^	1 (0.7)	62 (41.6)	20 (13.4)	43 (28.9)	15 (10.1)	8 (5.4)	149
12e	Difficulty chewing or swallowing ^	2 (1.3)	95 (63.8)	18 (12.1)	21 (14.1)	8 (5.4)	5 (3.4)	149
12f	Position (e.g., your posture, ease of access to food tray) ^	4 (2.7)	78 (53.1)	16 (10.9)	25 (17.0)	5 (3.4)	19 (12.9)	147
Staff Interactions and Assistance								
14a	Does the meal tray (including cutlery, serviettes, packaging etc.) have everything you need?	2 (1.3)	3 (2.0)	0	6 (4.0)	11 (7.4)	127 (85.2)	149
14b	Is assistance available if you need help opening the packaging on the meal tray?	10 (6.7)	1 (0.7)	1 (0.7)	6 (4.0)	10 (6.7)	121 (81.2)	149
14c	When you need help, are staff there to provide assistance at your mealtimes?	3 (2.0)	0	1 (0.7)	7 (4.7)	14 (9.4)	124 (83.2)	149
14d	Have the interactions you’ve had with staff during your mealtimes been positive?	0	3 (2.0)	2 (1.3)	7 (4.7)	22 (14.8)	115 (77.2)	149
Food Ordering System								
16a	Are the meals that you order from the menu the meals that you receive?	17 (11.5)	2 (1.4)	2 (1.4)	23 (15.5)	33 (22.3)	71 (48.0)	148
16b	Are the main meals served at an appropriate time for you?	4 (2.7)	9 (6.1)	5 (3.4)	17 (11.6)	21 (14.3)	91 (61.9)	147
16c	Are the staff who bring and take your menu friendly and polite?	12 (8.1)	3 (2.0)	0 (0.0)	6 (4.1)	9 (6.1)	118 (79.7)	148
Additional scale item		No *n* (%)	Sometimes *n* (%)	Yes *n* (%)				*n*
7	Overall, have your meals been enjoyable?	25 (16.8)	40 (26.8)	84 (56.4)				149

^ Negatively phrased items are reverse-coded.

## Data Availability

The data presented in this study are available on request from the corresponding author.

## Supplementary Materials

The following supporting information can be downloaded at: https://www.mdpi.com/article/10.3390/nu15122747/s1, File S1: The Austin Health Patient Mealtime Experience Tool.
