# Supplementary material for: Patient Mealtime Experience: Capturing Patient Perceptions Using a Novel Patient Mealtime Experience Tool
Source: Nutrients. 2023 Jun 14;15(12):2747. doi: 10.3390/nu15122747 (PMC10300684; doi:10.3390/nu15122747)
Supplement: Supplementary file 1 [file nutrients-15-02747-s001.zip › nutrients-2416720-supplementary.pdf]

Austin HEALTH

Patient Mealtime Experiences at Austin Health

This mealtime experience survey was developed following feedback from patients. This survey allows you the opportunity to share your opinion and experience of mealtimes during your admission at Austin Health.

By 'mealtime experiences' we mean:

- The environment (i.e. noise, comfort) in which you eat your meals
- The quality of food (including taste, presentation, temperature and variety)
- Interactions with staff and/or visitors
- Assistance provided during your mealtimes
- Food ordering system

1. How long have you been a patient at Austin Health? ____________________________

2. What is your current diet in the hospital? (e.g. regular, diabetic, gluten free, vegetarian, pureed)

____

3. Where do you eat your main meals in the hospital?

- ❑ Shared dining room
- ❑ Individual room/bed area
- ❑ Shared room/bed area

4. Please rate how you're feeling today

Low ☆ ☆ ☆ ☆ ☆ Great

5. Why?

____

Food Quality

6. For each of the following questions, please select one answer.

a) How frequently have you been satisfied with the quality of the food you have received at Austin Health?

Always Often Sometimes Rarely Never Not Applicable

b) Have the meals offered been appropriate for your beliefs or needs? (e.g. religious, cultural, vegan)?

Always Often Sometimes Rarely Never Not Applicable

c) Has there been variety in your meal choices?

Always Often Sometimes Rarely Never Not Applicable

d) Has the serving size of your meals been adequate?

Always Often Sometimes Rarely Never Not Applicable

e) Have the meals been served at a suitable temperature?

Always Often Sometimes Rarely Never Not Applicable

f) Have the meals looked appetising when they were presented?

Always Often Sometimes Rarely Never Not Applicable

g) Has the taste and flavour of the meals been to your liking?

Always Often Sometimes Rarely Never Not Applicable

7. Overall, have your meals been enjoyable?

- ☐ Yes
- ☐ No
- ☐ Sometimes

8. Why/Why not?

____

____

9. Further comments regarding the quality of food:

____

____

Environment

The following questions relate to the environment/space/room in which you sit and eat your meals. For each of the following questions, please circle one answer.

10. Do the following factors affect the amount of food you eat during mealtimes?

a) Noise

Always Often Sometimes Rarely Never Not Applicable

b) Visitors and/or other patients

Always Often Sometimes Rarely Never Not Applicable

c) Room surroundings (e.g. layout of the room, furniture, lighting, ambience)

Always Often Sometimes Rarely Never Not Applicable

d) Interruptions by hospital staff (e.g. wanting to speak to you or give you treatment)

Always Often Sometimes Rarely Never Not Applicable

e) Smells and odours

Always Often Sometimes Rarely Never Not Applicable

11. Please provide any further comments regarding your mealtime environment:

____

____

12. Do the following aspects affect the amount of food you eat during mealtimes?

a) Loss of appetite

Always Often Sometimes Rarely Never Not Applicable

b) Nausea and/or vomiting

Always Often Sometimes Rarely Never Not Applicable

c) Pain

Always Often Sometimes Rarely Never Not Applicable

d) Tiredness

Always Often Sometimes Rarely Never Not Applicable

e) Difficulty chewing or swallowing

Always Often Sometimes Rarely Never Not Applicable

f) Position (e.g. your posture, ease of access to food tray)

Always Often Sometimes Rarely Never Not Applicable

13. Please specify any other factors affecting the amount of food you eat during mealtimes?

____

____

Staff Interactions/Assistance

14. For each of the following questions, please select one answer.

a) Does the meal tray (including cutlery, serviettes, packaging etc.) have everything you need?

Always Often Sometimes Rarely Never Not Applicable

b) Is assistance available if you need help opening the packaging on the meal tray?

Always Often Sometimes Rarely Never Not Applicable

c) When you need help, are staff there to provide assistance at your mealtimes?

Always Often Sometimes Rarely Never Not Applicable

d) Have the interactions you've had with staff during your mealtimes been positive?

Always Often Sometimes Rarely Never Not Applicable

15. Please provide any further comments about your staff interactions/assistance:

____

____

Food Ordering System

16. For each of the following questions, please select one answer.

a) Are the meals that you order from the menu the meals that you receive?

Always Often Sometimes Rarely Never Not Applicable

b) Are the main meals served at an appropriate time for you?

Always Often Sometimes Rarely Never Not Applicable

c) Are the staff who bring and take your menu friendly and polite?

Always Often Sometimes Rarely Never Not Applicable

17. Please provide any further comments about ordering your meals:

____

____

Short Answer

18. Please provide any further comments about your mealtime experience at Austin Health:

____

____

____

19. Did you require assistance from staff with completing this survey?

- ❑ Yes
- ❑ No
